# Phase Stability and Magnetic Properties of Compositionally Complex *n* = 2 Ruddlesden–Popper Perovskites

**DOI:** 10.1021/acs.inorgchem.3c04277

**Published:** 2024-04-03

**Authors:** Rebecca Clulow, Prativa Pramanik, Amanda Stolpe, Deep C. Joshi, Roland Mathieu, Paul F. Henry, Martin Sahlberg

**Affiliations:** † Department of Chemistry - Ångström Laboratory, Uppsala University, Box 538, 751 21 Uppsala, Sweden; ‡ Department of Materials Science and Engineering, Uppsala University, Box 35, 751 03 Uppsala, Sweden; § FSCN Research Centre, Surface and Colloid Engineering, Mid Sweden University, 851 70 Sundsvall, Sweden; ∥ ISIS Pulsed Neutron & Muon Facility, Rutherford Appleton Laboratory, Harwell Campus, Didcot OX11 0QX, United Kingdom

## Abstract

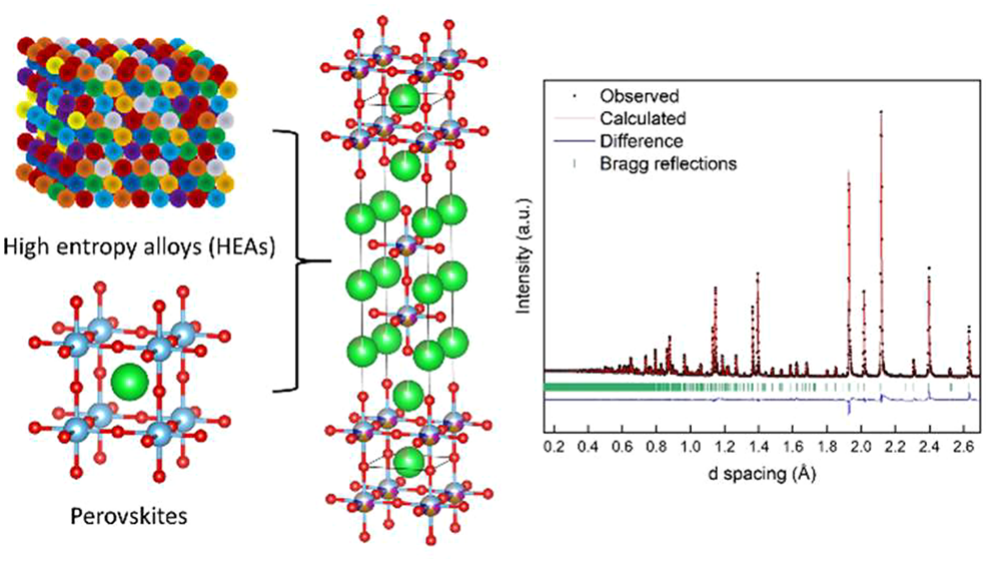

Four new compositionally complex perovskites with multiple (four or more) cations on the B site of the perovskites have been studied. The materials have the general formula La_0.5_Sr_2.5_(M)_2_O_7−δ_ (M = Ti, Mn, Fe, Co, and Ni) and have been synthesized via conventional solid-state synthesis. The compounds are the first reported examples of compositionally complex *n* = 2 Ruddlesden–Popper perovskites. The structure and properties of the materials have been determined using powder X-ray diffraction, neutron diffraction, energy dispersive X-ray spectroscopy, X-ray photoelectron spectroscopy, and magnetometry. The materials are isostructural and adopt the archetypal *I*4/*mmm* space group with the following unit cell parameters: *a* ∼ 3.84 Å, and *c* ∼ 20.1 Å. The measured compositions from energy dispersive X-ray spectroscopy were La_0.51(2)_Sr_2.57(7)_Ti_0.41(2)_Mn_0.41(2)_Fe_0.39(2)_Co_0.38(1)_Ni_0.34(1)_O_7−δ_, La_0.59(4)_Sr_2.29(23)_Mn_0.58(5)_Fe_0.56(6)_Co_0.55(6)_Ni_0.42(4)_O_7−δ_, La_0.54(2)_Sr_2.49(13)_Mn_0.41(2)_Fe_0.81(5)_Co_0.39(3)_Ni_0.36(3)_O_7−δ_, and La_0.53(4)_Sr_2.55(19)_Mn_0.67(6)_Fe_0.64(5)_Co_0.31(2)_Ni_0.30(3)_O_7−δ_. No magnetic contribution is observed in the neutron diffraction data, and magnetometry indicates a spin glass transition at low temperatures.

## Abstract 2

Compositionally complex/high-entropy materials constitute a new and promising area of research, the materials are known to have improved properties compared to those of conventional materials and offer a new level of tunability. Four new compositionally complex *n* = 2 Ruddlesden−Popper perovskites have been synthesized and studied by neutron diffraction, X-ray diffraction, magnetometry, energy dispersive X-ray spectroscopy, and X-ray photoelectron spectroscopy. The materials have no significant phase segregation and exhibit a spin glass transition at low temperatures.

## 1 Introduction

**Figure 1:**
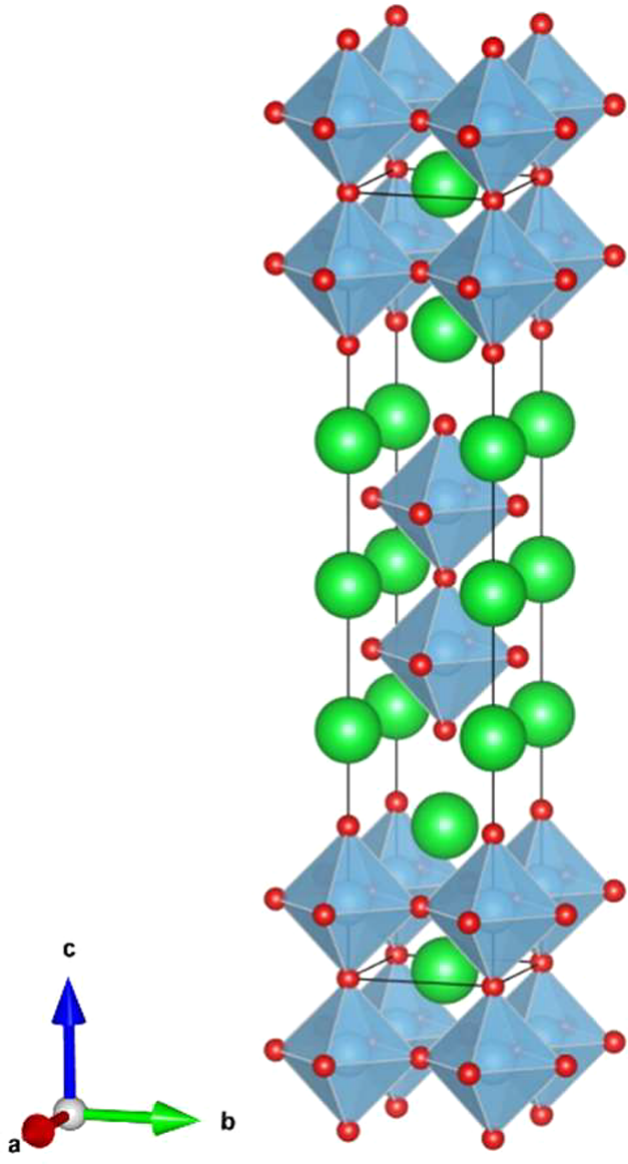
Crystal structure of an *n* = 2 Ruddlesden–Popper perovskite (A_3_B_2_O_7_). A site cations are colored green, B site cations blue, and oxygen atoms red.

Perovskite oxides (ABO_3_) are one of the most widely studied structure types in solid-state chemistry and display a wide range of functional properties, including electronic and magnetic properties.^1−4^ Their wide range of functionalities is largely due to the versatility of the structure type that can accommodate almost every element in the periodic table in its structure, allowing a wide scope for variation, modification, and optimization. Typically, the A site contains a larger cation from either group I, II or lanthanoids. While the B site cation is typically smaller, often including the 3d, 4d, and 5d elements, other elements and ions can be incorporated leading to a large range of possible compositions. In addition to the large number of possible cations, there are several layered forms of perovskites, notably the Ruddlesden–Popper type with the general formula A_*n*+1_B_*n*_O_3*n*+1_. The structure is composed of *n* ABO_3_ perovskite layers separated by rock salt layers as shown in Figure 1. The rock salt layers are composed of an alternating checkerboard arrangement of the A site cations and oxygens. The compounds also have an interesting range of properties for ferroelectrics, magnetism, and thermal expansion.^5−7^ Given the flexibility in compositions, there have been numerous studies investigating the effects of composition on the properties; however, thus far, these have been predominantly limited to compositions with two or three cations, and there has been little investigation of more complex high-entropy type compositions that include four or more cations on the same crystallographic position. The increase in the degree of compositional control leads to more tunable properties and could allow for materials with multiple functionalities, which are significant advantages over more conventional materials.

High-entropy alloys have attracted a significant amount of research interest in recent years, the materials incorporate multiple principle elements and were first reported by Yeh et al.^8^ The high-entropy alloys were found to have improved hydrogen storage and tensile strength.^9,10^ The approach has more recently been expanded to include ceramics and oxides.^11−13^ As a result, there have now been several studies regarding compositionally complex and high-entropy perovskites, particularly those with the ABO_3_ type structure.^12−15^ The compositionally complex *n* = 1 Ruddlesden–Popper perovskites have only more recently been reported with research largely focused on the cuprate and thin film-based materials.^16−19^ However, a more recent study investigated the local ordering of the cations in a compositionally complex *n* = 1 Ruddlesden–Popper perovskite for the first time.^20^ These types of materials display a wide range of functional properties, including ionic conductivity, magnetism, and ferroelectricity.^14,21,22^ The magnetic properties of ABO_3_ type compositionally complex perovskites have generally been antiferromagnetic in nature with a small ferrimagnetic moment as observed in both the RE(M5)O_3_ and (RE5)MO_3_, where RE are rare earth elements (Gd, La, Nd, Sm, and Y) and M are transition metals (Co, Cr, Fe, Mn, and Ni).^13,14,23^ A similar effect was recently reported in a study focusing on the Sm(M4)O_3_ compounds where M = Co, Cr, Fe, and Mn. The difference in zero-field and field-cooled magnetization data is often attributed to glassy behavior without further investigation but is more likely due to the inhomogeneous nature of the samples.^24^

The compounds presented in this paper are based on the *n* = 2 Ruddlesden–Popper perovskites with a general formula of La_0.5_Sr_2.5_(M)_2_O_7_, where M is a combination of Ti, Mn, Fe, Co, and Ni. The elements were chosen on the basis of atomic size and to allow the investigation of the magnetic behavior of the system. The phase stability and magnetic properties of the system are reported herein. The results represent the first reported examples of compositionally complex *n* = 2 Ruddlesden–Popper type perovskites.

## 2 Experimental Section

No uncommon hazards were noted during the synthesis and characterization of the reported compounds.

### 2.1 Sample Preparation

Samples were prepared by using conventional solid-state synthesis using commercially available reagents. La_2_O_3_ (Highways International), SrCO_3_ (Cerac), TiO_2_ (Merck), MnO_2_ (Cerac), Fe_2_O_3_ (Sigma-Aldrich), NiO (Sigma-Aldrich), and Co (Höganas) are all >99% pure. Co was oxidized to Co_3_O_4_ at 600 °C for 6 h, and its purity confirmed by powder X-ray diffraction prior to further synthesis. La_2_O_3_ was predried at 975 °C prior to weighing. The reagents were mixed in a mortar and pestle with ethanol before being pressed into pellets and heated in alumina crucibles at 1000 °C for 12 h and 1400 °C for 36 h in air with intermediate grinding using a heating/cooling rate of 5 °C/min.

### 2.2 X-ray Diffraction

Powder X-ray diffraction patterns were measured on a Bruker D8 ADVANCE diffractometer equipped with a Lynx-eye XE position sensitive detector (PSD) using Cu Kα radiation (λ = 1.5418 Å). Diffraction patterns were measured in the 2θ range of 10–100° using a step size of 0.014°. The data were analyzed using the Rietveld refinement^25^ method within Topas 6.^26^ The refined parameters include the zero point, background, unit cell, peak shape, and atomic positions, while the occupancies were fixed at their nominal values.

### 2.3 Neutron Diffraction

Neutron diffraction patterns were measured at the ISIS neutron and muon source using the Polaris time-of-flight diffractometer.^27^ The data were collected on ∼3 g of sample in 6 mm vanadium cans. Data reduction and generation of files suitable for refinement used the Mantid open source software.^28^ A second set of neutron diffraction data was also collected using the MEREDIT instrument at the Nuclear Physics Institute in Rez, Czech Republic. The measurements utilized a copper mosaic monochromator (reflection 220) with a wavelength of 1.46 Å in the 2θ range of 4–144°. The data were analyzed using the Rietveld refinement method using GSAS.^25,29^

### 2.4 Energy Dispersive X-ray Spectroscopy (EDS)

Data were collected using a Zeiss Leo 1550 field emission scanning electron microscopy equipped with an Aztec dispersive X-ray detector on a minimum of 10 points per sample using an accelerating voltage of 20 kV. Pellets of sintered powder were attached to conducting carbon tape for point analysis and mapping.

### 2.5 X-ray Photoelectron Spectroscopy (XPS)

Data were collected by using an Ulvac-Phi Quantera II spectrometer with monochromated Al Kα radiation (1486.7 eV). During measurements, a takeoff angle of 45° and a spot size of 100 μm were used. The spectra were measured on sintered and polished pellets of the samples under constant charge neutralization with an electron flood gun and low-energy Ar^+^ ions. The sample underwent presputtering to remove adsorbed species using a 200 eV Ar^+^ beam for 20 min. The position of the C 1s peak from adventitious carbon before sputtering (285.0 eV) was used as the charge reference. Survey and core level spectra for each element were recorded. Peak fitting of the La 3d_5/2_, Sr 3d, and O 1s peaks was performed using the Multipak software with a Shirley background and Voigt peak shapes with an 80% Gaussian contribution.

### 2.6 Magnetometry

The dc magnetization and ac susceptibility measurements were performed by using a SQUID magnetometer from Quantum Design Inc. The temperature dependence of magnetization *M*(*T*) was recorded in zero-field-cooled (ZFC) and field-cooled (FC) conditions under the application of a constant magnetic field *H* of 25 and 1000 Oe. ZFC memory experiments^30^ were also performed by recording the temperature-dependent ZFC magnetization on reheating after including halts in the cooling to the lowest temperature. Magnetic hysteresis curves *M*(*H*) were recorded at a constant temperature *T* of 5 K with magnetic fields swept between −50 and 50 kOe. ac susceptibility χ(ω = 2π*f*, *T*) data were collected using an ac excitation *h* = 4 Oe for frequencies (*f*) of 1.7, 17, and 170 Hz.

## 3 Results and Discussion

### 3.1 Phase Analysis

**Figure 2:**
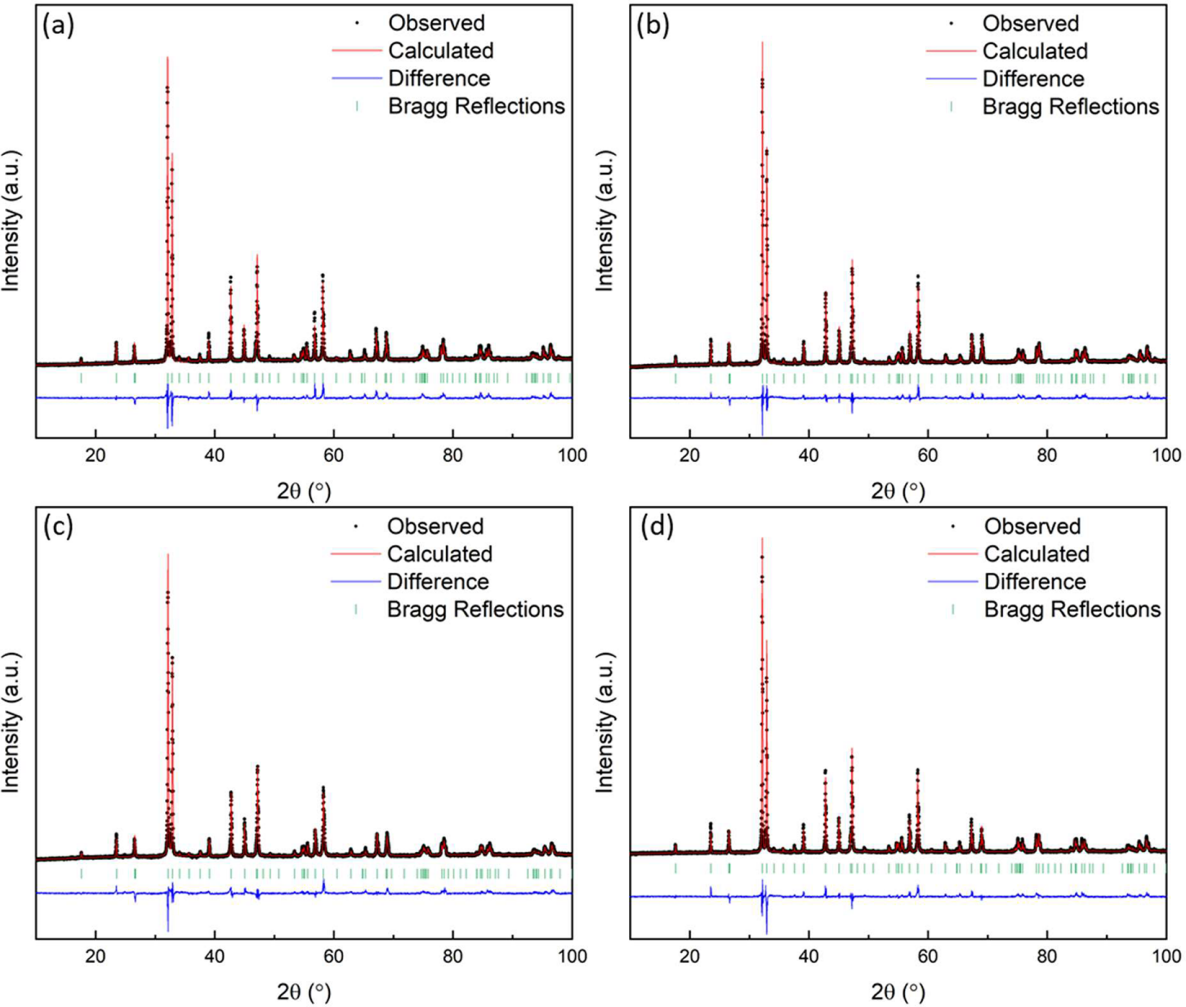
Powder X-ray diffraction patterns (λ = 1.5418 Å) of (a) La_0.5_Sr_2.5_(Ti_1/5_Mn_1/5_Fe_1/5_Co_1/5_Ni_1/5_)_2_O_7−δ_, (b) La_0.5_Sr_2.5_(Mn_1/4_Fe_1/4_Co_1/4_Ni_1/4_)_2_O_7−δ_, (c) La_0.5_Sr_2.5_(Mn_1/5_Fe_2/5_Co_1/5_Ni_1/5_)_2_O_7−δ_, and (d) La_0.5_Sr_2.5_(Mn_2/6_Fe_2/6_Co_1/6_Ni_1/6_)_2_O_7−δ_.

The four new La_0.5_Sr_2.5_(M)_2_O_7−δ_ (M = Ti, Mn, Fe, Co, and Ni) compounds were studied using powder X-ray diffraction and Rietveld refinement revealing the prototype Sr_3_Ti_2_O_7_ type structure with the *I*4/*mmm* space group and the following unit cell parameters: *a* ∼ 3.85 Å, and *c* ∼ 20.10 Å. The results of the Rietveld refinements are shown in Figure 2 and Table 1, revealing a high level of purity in the samples and a good fit to the structural model, although the compounds exhibited a noticeable preferred orientation in the 00*l* direction. The refined atomic coordinates are listed in Table S1. The structure of La_0.5_Sr_2.5_(Mn_1/4_Fe_1/4_Co_1/4_Ni_1/4_)_2_O_7−δ_ was also investigated using low-temperature X-ray diffraction but did not reveal evidence of a phase change at low temperature. While examples of compositionally complex ABO_3_ and *n* = 1 perovskites have previously been reported in the literature, to the best of our knowledge these are the first reported examples of compositionally complex *n* = 2 Ruddlesden–Popper perovskites

**Table 1:** Results of Rietveld Refinements of La_0.5_Sr_2.5_M_2_O_7−δ_ Compounds from Powder X-ray Diffraction Data

	*a* (Å)	*c* (Å)	*R*_wp_	χ^2^
La_0.5_Sr_2.5_(Ti_1/5_Mn_1/5_Fe_1/5_Co_1/5_Ni_1/5_)_2_O_7−δ_	3.8551(1)	20.1646(4)	7.51	5.57
La_0.5_Sr_2.5_(Mn_1/4_Fe_1/4_Co_1/4_Ni_1/4_)_2_O_7−δ_	3.8438(1)	20.0971(3)	5.81	4.45
La_0.5_Sr_2.5_(Mn_1/5_Fe_2/5_Co_1/5_Ni_1/5_)_2_O_7−δ_	3.8490(1)	20.1211(4)	6.44	4.39
La_0.5_Sr_2.5_(Mn_2/6_Fe_2/6_Co_1/6_Ni_1/6_)_2_O_7−δ_	3.8465(1)	20.1215(3)	7.28	4.41

The composition of the samples was further analyzed using EDS analysis. The measurements were carried out on sintered pellets of each of the samples giving compositions within 4 atom % of the nominal compositions as summarized in Table 2. Though a slight Ni deficiency is evident in many of the compositions, full details of all measurements are listed in Tables S2–S5. EDS mapping was also carried out on a sintered pellet of La_0.5_Sr_2.5_(Ti_1/5_Mn_1/5_Fe_1/5_Co_1/5_Ni_1/5_)_2_O_7−δ_, revealing good homogeneity of all elements across the area investigated as shown in Figures S1 and S2.

**Table 2:** Nominal and Measured Compositions of the Four New Compounds, as Determined from EDS Measurements

nominal composition	measured composition
La_0.5_Sr_2.5_Ti_0.4_Mn_0.4_Fe_0.4_Co_0.4_Ni_0.4_O_7−δ_	La_0.51(2)_Sr_2.57(7)_Ti_0.41(2)_Mn_0.41(2)_Fe_0.39(2)_Co_0.38(1)_Ni_0.34(1)_O_7−δ_
La_0.5_Sr_2.5_Mn_0.5_Fe_0.5_Co_0.5_Ni_0.5_O_7−δ_	La_0.59(4)_Sr_2.29(23)_Mn_0.58(5)_Fe_0.56(6)_Co_0.55(6)_Ni_0.42(4)_O_7−δ_
La_0.5_Sr_2.5_Mn_0.4_Fe_0.8_Co_0.4_Ni_0.4_O_7−δ_	La_0.54(2)_Sr_2.49(13)_Mn_0.41(2)_Fe_0.81(5)_Co_0.39(3)_Ni_0.36(3)_O_7−δ_
La_0.5_Sr_2.5_Mn_0.67_Fe_0.67_Co_0.33_Ni_0.33_O_7−δ_	La_0.53(4)_Sr_2.55(19)_Mn_0.67(6)_Fe_0.64(5)_Co_0.31(2)_Ni_0.30(3)_O_7−δ_

In addition to the four new compounds presented, the effects of A site substitution and various compositions on the B site on phase stability were also investigated. The flexibility of the A site of the material was investigated through the substitution of the Sr with the group II metals Ca^2+^ and Ba^2+^, though no compounds containing Ca or Ba could be synthesized in this structure family. The effect of the La_3–*x*_Sr_*x*_ solid solution was also studied, revealing a relatively narrow stability window in the region of *x* = 2.50–2.75, depending on the B site composition and charge balance. Compositions outside of this range typically contained additional A_2_BO_4_/ABO_3_ type phases.

### 3.2 Neutron Diffraction Data

**Figure 3:**
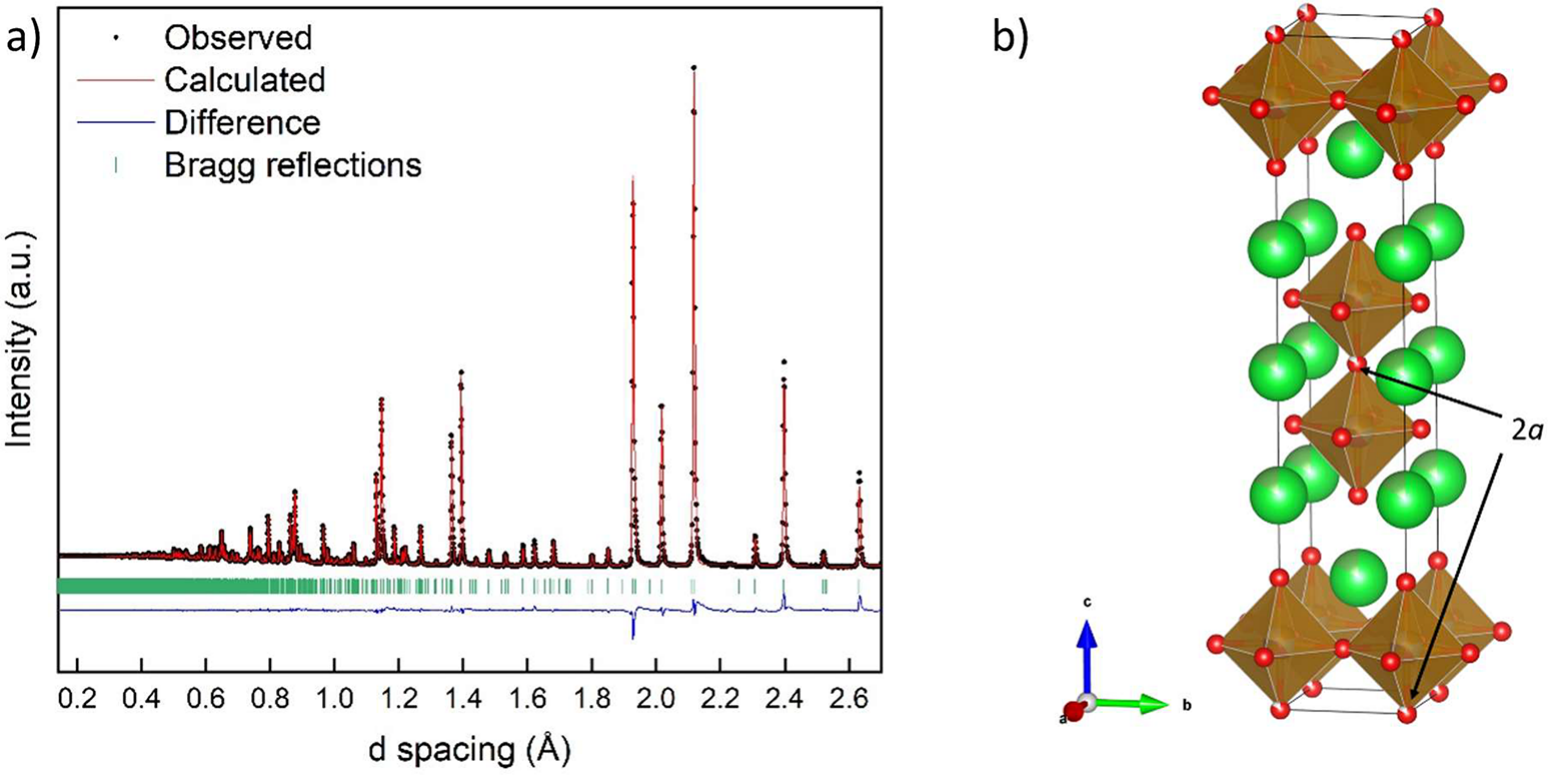
(a) Powder neutron diffraction patterns and Rietveld refinement of La_0.5_Sr_2.5_(Ti_1/5_Mn_1/5_Fe_1/5_Co_1/5_Ni_1/5_)_2_O_7−δ_ (*R*_wp_ = 2.16; χ^2^ = 8.33) from bank 5 data from the Polaris time-of-flight diffractometer at the ISIS neutron and muon source and (b) crystal structure of La_0.5_Sr_2.5_(Ti_1/5_Mn_1/5_Fe_1/5_Co_1/5_Ni_1/5_)_2_O_7−δ_ derived from powder neutron diffraction data: green for La and Sr, brown for transition metals, and red for oxygen.

La_0.5_Sr_2.5_(Ti_1/5_Mn_1/5_Fe_1/5_Co_1/5_Ni_1/5_)_2_O_7−δ_ and La_0.5_Sr_2.5_(Mn_1/4_Fe_1/4_Co_1/4_Ni_1/4_)_2_O_7−δ_ underwent further analysis by neutron diffraction to investigate the formation of oxygen vacancies and cation ordering at room temperature. The diffraction pattern and Rietveld refinement of La_0.5_Sr_2.5_(Ti_1/5_Mn_1/5_Fe_1/5_Co_1/5_Ni_1/5_)_2_O_7−δ_ are shown in Figure 3a with further details provided in Table 3 and Figure S3. The results are consistent with the X-ray diffraction data with the following refined cell parameters: *a* = 3.8535(1) Å, and *c* = 20.1539(2) Å. Both X-ray and neutron diffraction data suggest that there is no significant phase segregation in the sample. However, neutron diffraction does reveal evidence of a minor secondary ABO_3_ type phase evident in the peak at ∼2.23 Å. The broadening at 2.13 Å can be attributed to the vanadium sample holder. The neutron diffraction data did not reveal evidence of any long-range cation ordering, though further total scattering experiments could provide further insight on any short-range order. The occupancies of both the A site and compositionally complex B site were kept fixed at their nominal compositions, while the oxygen site occupancies were refined to determine the presence of oxygen vacancies. The oxygen 2*a* site had a refined occupancy of 0.822(8) (Table 3 and Figure 3b), giving a formula of La_0.5_Sr_2.5_(Ti_1/5_Mn_1/5_Fe_1/5_Co_1/5_Ni_1/5_)_2_O_6.82_ and an average B site oxidation state of approximately +3.6. The occupancies of the oxygen 4*e* and 8*g* sites were allowed to be refined but did not vary from 1.0. This finding is consistent with research on conventional *n* = 2 Ruddlesden–Popper perovskites that are known to accommodate oxygen vacancies at the 2*a* position.^31−33^ This feature has also been investigated in the La_0.54_Sr_2.46_Fe_2_O_7−δ_ system via powder diffraction and density functional theory methods. The results revealed a significantly lower oxygen vacancy formation energy for the 2*a* position in comparison to that of the 4*e* or 8*g* site, though a small number of vacancies have been observed on the 8*g* site at increased temperatures (800–900 °C).^34^ A second set of neutron diffraction data for the La_0.5_Sr_2.5_(Ti_1/5_Mn_1/5_Fe_1/5_Co_1/5_Ni_1/5_)_2_O_7−δ_ compound was previously collected using the MEREDIT instrument, and the results are consistent with the data from Polaris and are shown in full in Figure S4 and Table S6. The second sample, La_0.5_Sr_2.5_(Mn_1/4_Fe_1/4_Co_1/4_Ni_1/4_)_2_O_7−δ_, was also analyzed using neutron diffraction. The data are consistent with an *n* = 2 Ruddlesden–Popper perovskite, and there was no significant phase segregation; however, small amounts of an ABO_3_ phase and NiO were observed. The data were consistent with the Ti-containing sample, suggesting an oxygen deficiency in the materials localized predominantly on the O 2*a* site with a refined occupancy of ∼0.72 (Table S7). However, the data could not be satisfactorily fitted using the *I*4/*mmm* model, suggesting additional ordering in the sample that will form the basis of a follow-up study. The fit to the *I*4/*mmm* model is shown in Figure S5.

**Table 3:** Refined Atomic Positions and Occupancies of La_0.5_Sr_2.5_(Ti_1/5_Mn_1/5_Fe_1/5_Co_1/5_Ni_1/5_)_2_O_7−δ_ Derived from Powder Neutron Diffraction Data Collected at Room Temperature

atom	Wyckoff position	*x*	*y*	*z*	occupancy
La	2*b*	0	0	0.5	1/6
Sr	2*b*	0	0	0.5	5/6
La	4*e*	0	0	0.31754(3)	1/6
Sr	4*e*	0	0	0.31754(3)	5/6
M	4*e*	0	0	0.09953(6)	1
O1	8*g*	0	0.5	0.09344(4)	1
O2	4*e*	0	0	0.19474(4)	1
O3	2*a*	0	0	0	0.822(8)

### 3.3 Valence-State Determination

**Figure 4:**
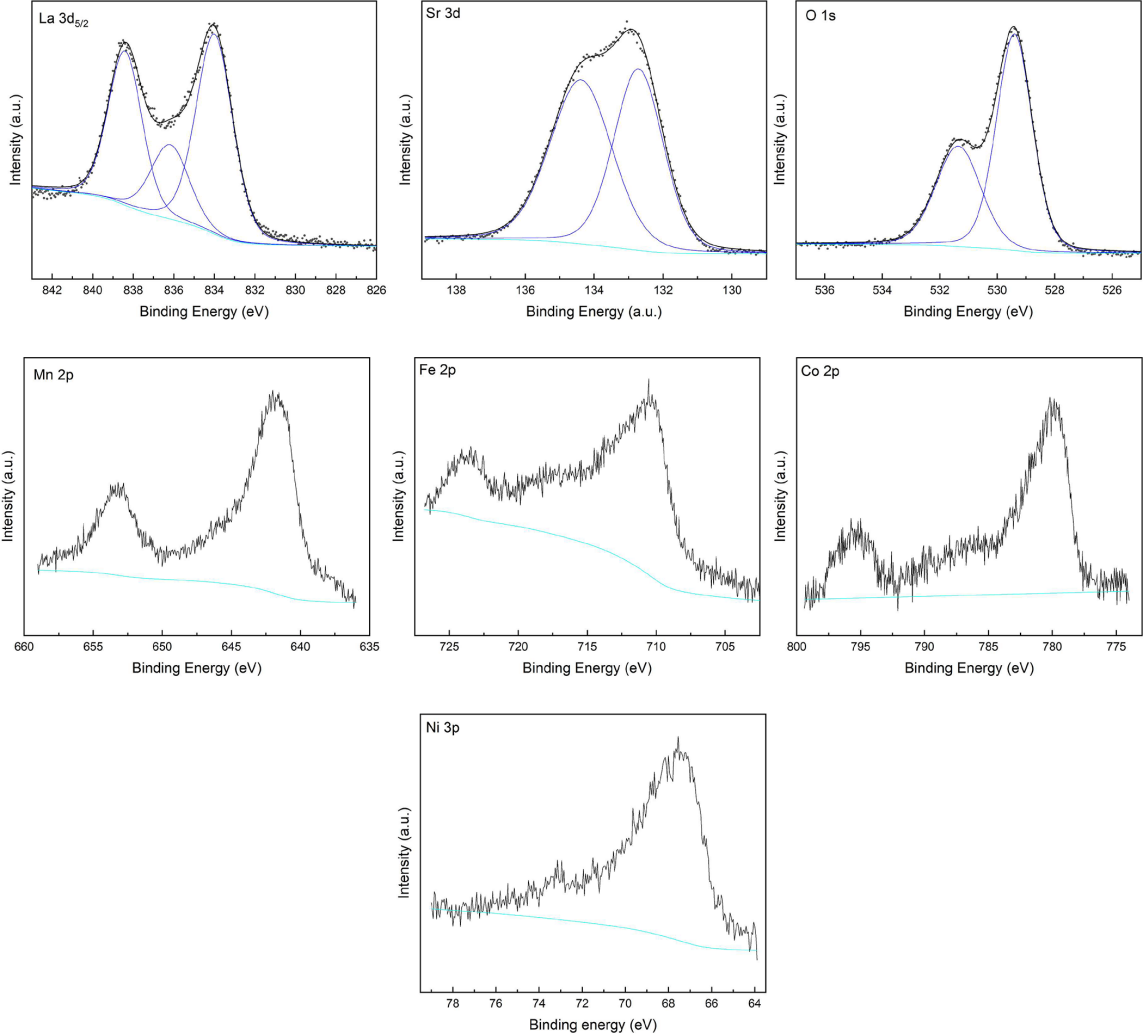
XPS spectra for a La_0.5_Sr_2.5_(Mn_1/4_Fe_1/4_Co_1/4_Ni_1/4_)_2_O_7−δ_ sample. Raw data are plotted as gray circles. The results of peak fitting are colored black. Individual peaks are colored blue. Background is colored light blue.

An average B site oxidation state was estimated from the neutron diffraction data; however, XPS was used to investigate the oxidation states of each of the elements in the La_0.5_Sr_2.5_(Mn_1/4_Fe_1/4_Co_1/4_Ni_1/4_)_2_O_7−δ_ sample. However, it should be noted that the relatively small amounts of each of the transition metals (∼4 atom %), overlapping Auger bands, and the overlap of the Ni 2p band with the La 3d region make unambiguous assignment and quantification of oxidation states challenging for these types of materials. Survey spectra of the sample were measured pre- and postsputtering as shown in Figure S6, with the regions used for more detailed analysis highlighted. Core level spectra of each of the elements were recorded, as shown in Figure 4. The various charge references used in the literature and the variation of the adventitious carbon 1s binding energy do not allow for direct comparison of the absolute binding energies; however, a more qualitative analysis of the spectral features can be undertaken.^35^

The La 3d_5/2_ region contains a double peak with maxima at ∼834 and 838 eV, which corresponds to the values and splitting observed in both La_2_O_3_ and the La_1–*x*_Sr_*x*_MnO_3_ compounds.^36−38^ The Sr 3d spectrum was fitted with two peaks with values consistent with the literature for Sr^2+^ in perovskite compounds.^37^ The measured O 1s spectrum contained two peaks at ∼531.5 and 529 eV, indicating two different oxygen environments as often observed in oxide materials.^13,31,39^ The valence states of the transition metals were also investigated using XPS; care was taken to minimize reduction of the oxidation states during Ar^+^ sputtering by using a low sputtering voltage of 200 eV, but it will always be present to some extent. The Mn 2p peak contained two maxima at ∼642 eV (2p_3/2_) and 653 eV (2p_1/2_);^40−42^ the peak at ∼642 eV is asymmetric, which is a common feature in the XPS spectra of transition metals and does not allow for unambiguous assignment of the Mn oxidation state. The measurement of standard samples during the experiment would be required for a more definitive assignment of the valence states. However, the characteristic satellite peak at 647 eV associated with the Mn^2+^ state is missing from the spectrum.^43^ Hence, the expected oxidation states are likely similar to those observed in the La_1–*x*_Sr_*x*_MnO_3_ compounds with a mixture of Mn^3+^ and Mn^4+^ states.^37,44^ The Fe 2p spectrum contained two maxima at ∼724 eV (2p_1/2_) and 710 eV (2p_3/2_).^40,45^ A satellite feature is visible at ∼718.5 eV, which is characteristic of the Fe^3+^ oxidation state rather than Fe^2+^, which typically has a lower binding energy in the region of ∼715 eV, though comparison of absolute values is challenging due to the variation in charge references.^40,45,46^ This is consistent with reports of a mixed 3+/4+ oxidation state observed in the Sr_3_Fe_2_O_7−δ_ compounds, which were synthesized under similar conditions.^31,39^ The Co 2p spectrum has peaks at ∼780 eV (2p_3/2_) and 796 eV (2p_1/2_);^40^ the values correspond well to the Co 2p literature values observed in both CoO and Co_3_O_4_.^47,48^ The satellite feature just below 790 eV is indicative of the +3 oxidation state, while the feature slightly above the peak at 780 eV could indicate the presence of Co^4+^.^40,49^ Further analysis of standard Co-containing materials would be required for a definitive determination of the oxidation states. Due to the significant overlap of the La 3d peaks with the Ni 2p region, the Ni 3p region was used as an alternative to investigate its valence states despite its significantly weaker relative intensity. The spectrum contained a single peak and a satellite feature. The data did not allow the differentiation of the Ni^2+^ and Ni^3+^ states, which differ only in their relative binding energies. However, the data are similar to the observed spectra of Ni^3+^ in the LaNiO_3_ compounds and are consistent with the analysis of other compositionally complex perovskites.^13,50^ Given that the synthesis was carried out in air rather than an O_2_ atmosphere, the compound likely contains a mixture of Ni^2+^ and Ni^3+^ oxidation states as previously reported in other Ni-containing perovskites.^51^

The analysis of the XPS data suggests oxidation states of La^3+^Sr^2+^Mn^3+/4+^Fe^3+/4+^Co^3+/2+^Ni^3+^O^2–^ in the La_0.5_Sr_2.5_(Mn_1/4_Fe_1/4_Co_1/4_Ni_1/4_)_2_O_7−δ_ composition, which are lower than expected considering the observed oxygen vacancies and charge balance. However, other factors, including reduction of the transition metals during the sputtering, and La/Sr A site vacancies could be contributing factors. The data also suggest higher oxidation states than typically expected for the Fe and Ni elements during syntheses in air under atmospheric conditions. However, partial oxidation of Fe to 4+ and Ni to 3+ has been observed in other perovskite-based systems synthesized under similar conditions.^39,50,52^ Neutron diffraction refinements were also carried out with a fixed oxygen occupancy assuming an average oxidation state of +3 on the B site but negatively affected the quality of the Rietveld refinement, supporting the refined values of the oxygen occupancies. Detailed analysis of the valence states of the other compositions was not undertaken, but similar results are expected with spectra containing multiple oxidation states that are challenging to resolve. In addition, given the high degree of compositional complexity in the materials, it is also likely that there are regions of different compositions with clustering of the different elements in the material, which should be investigated as part of the total scattering study proposed. The oxidation states present in the sample are directly linked to the ionic radii of the transition metals; however, due to the difficulties in quantifying the relative ratios of the different valence states, definitive values of the average ionic radii are unobtainable. However, the differences in radii would be relatively small in this system; assuming low-spin states for each of the elements, the average radii would vary between 0.555 Å for the Mn^4+^Fe^4+^Co^3+^Ni^3+^ state, 0.559 Å for the Mn^3+^Fe^3+^Co^3+^Ni^3+^ state, 0.618 Å for the Mn^3+^Fe^3+^Co^2+^Ni^2+^ state, and 0.603 Å using the oxidation states observed in the XPS measurement (Mn^3+/4+^Fe^3+/4+^Co^3+/2+^Ni^2+^).

### 3.4 Magnetometry

**Figure 5:**
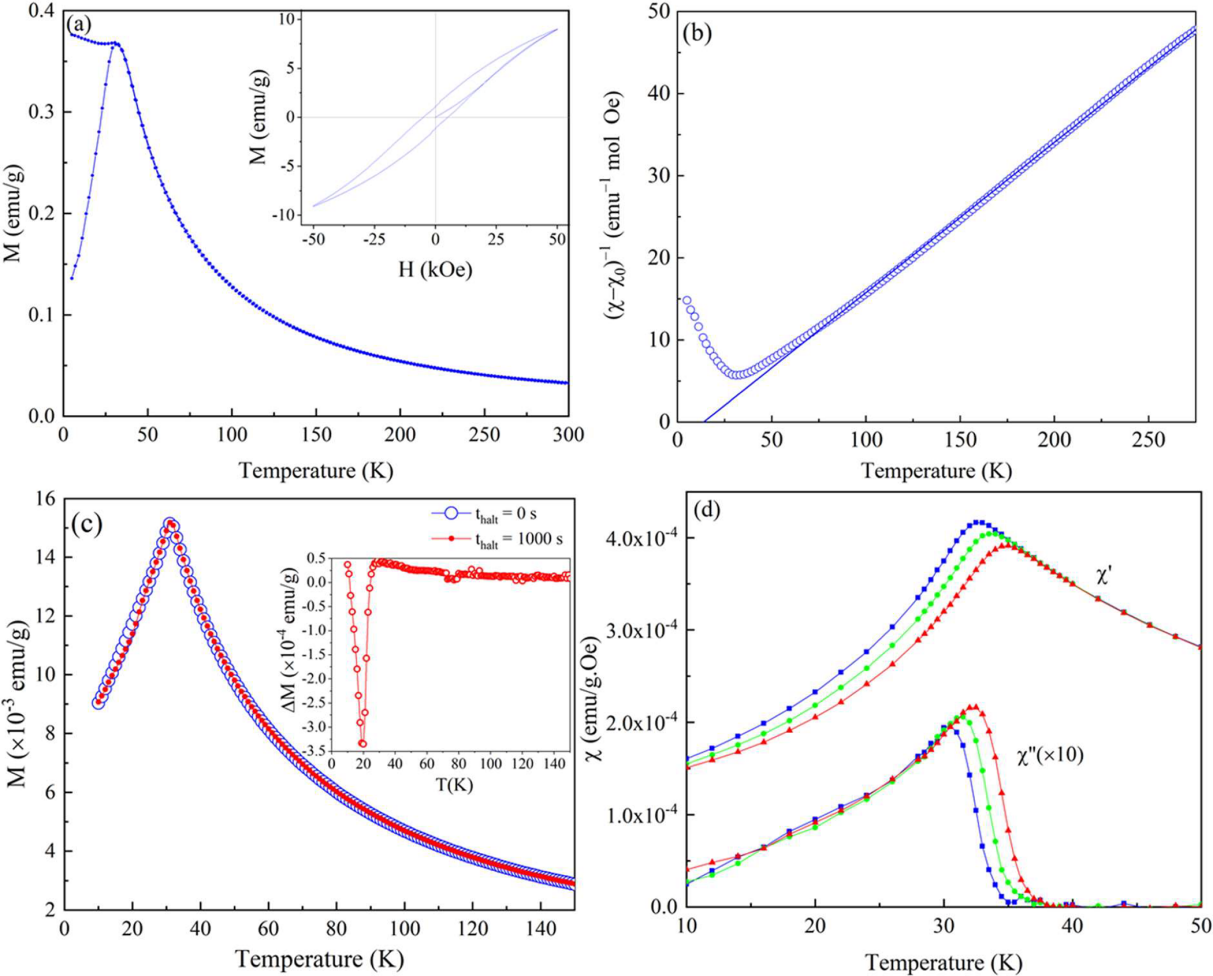
Magnetic behavior of La_0.5_Sr_2.5_(Mn_1/4_Fe_1/4_Co_1/4_Ni_1/4_)_2_O_7−δ_. (a) Temperature-dependent magnetization *M*(*T*) measured under ZFC and FC conditions with *H* = 1000 Oe. The inset shows *M* vs *H* recorded at a constant temperature of 5 K. (b) *M*(*T*) data replotted as 1/χ = *H*/*M* vs temperature for analysis of the Curie–Weiss behavior. A linear fit of the data is added. (c) ZFC memory plots. ZFC *M*(*T*) curves recorded under *H* = 25 Oe after cooling the sample from 300 K in zero magnetic field and making a halt at 20 K for a *t*_halt_ of 1000 s. The reference *M*(*T*) curve, i.e., without making any halt at 20 K, is also plotted. The corresponding Δ*M*(*T*) plot where Δ*M*(*T*) = *M*(*t*_halt_ = 1000 s) – *M*(*t*_halt_ = 0 s) is plotted in the inset. (d) Temperature dependence of the in-phase (χ′) and out-of-phase (χ″) components of the ac susceptibility recorded for three frequencies (170, 17, and 1.7 Hz); ac excitation *h* = 4 Oe.

The neutron diffraction data of the materials could be refined solely with a nuclear component, suggesting that the materials are in a paramagnetic state at room temperature. Magnetometry was employed to investigate the magnetic properties. The four samples were found to exhibit qualitatively and quantitatively similar magnetic behavior, which is illustrated in Figure 5 for La_0.5_Sr_2.5_(Mn_1/4_Fe_1/4_Co_1/4_Ni_1/4_)_2_O_7−δ_. See Figure S7 for a comparison of the magnetic properties of the four compositions. The ZFC *M*(*T*) data (Figure 5a) show a cusp below 32 K. Below that temperature, ZFC and FC curves show irreversibility and the FC magnetization curve increases only slightly as the temperature decreases. The observed behavior is reminiscent to that of spin glasses.^30^Figure 5a (inset) shows the magnetic field-dependent *M*(*H*) curve recorded at 5 K, which does not become saturated in the highest applied magnetic field. The ZFC magnetization data recorded at *H* = 1000 Oe are replotted as the inverse susceptibility 1/χ = *H*/*M* as a function of the temperature in Figure 5b. The inverse susceptibility follows a linear behavior with *T* down to the lowest temperature above the cusp, implying a Curie–Weiss behavior for the susceptibility, χ – χ_0_ = *C*/(*T* – θ_CW_) with a small, positive θ_CW_ = 14.5 K and effective magnetic moment μ_eff_ = 6.6 μ_B_/formula unit; χ_0_ ∼ −4 × 10^–3^ emu mol^–1^ Oe^–1^. The potential spin glass behavior^53^ may be investigated using “ZFC memory experiments”,^30,53^ as illustrated in Figure 5c. The reference curve is the typical temperature-dependent ZFC magnetization curve recorded upon reheating after cooling the sample in zero magnetic field from 300 K down to the lowest temperature (*T*_min_) of 10 K, whereas for the memory curve, a halt with a duration (*t*_halt_) of 1000 s was included at a *T*_halt_ of 20 K, i.e., below the cusp temperature, during the initial cooling (and, as for the reference, subsequently recording the ZFC magnetization on reheating from *T*_min_). The *M*(*T*) memory curve is completely identical and coincides with the reference curve except near *T*_halt_, where a dip was observed. This dip across *T*_halt_ is clearer in the Δ*M*(*T*) difference plot [where Δ*M*(*T*) = *M*(*t*_halt_ = 1000 s) – *M*(*t*_halt_ = 0 s)] shown in the inset. This is a typical behavior of spin glass that “remembers” its aging during the halt (memory effect) and “forgets” it (rejuvenation effect), if it is subjected to temperature changes.^30,53^ As shown in Figure 5d, the temperature dependence of the ac susceptibility is typical of that observed in spin glasses, with frequency-dependent cusps and onsets in the in-phase component χ′(*f*, *T*) and out-of-phase component χ″(*f*, *T*), respectively.^54^ Interestingly, the χ′(*f*, *T*) curves display no frequency dependence above the cusp temperature, and a sharp temperature onset is observed for χ″(*f*, *T*). This suggests that the material homogeneously undergoes a phase transition to a spin glass phase at low temperatures.^54,55^ The occurrence of such a phase transition is investigated in detail in ref (56). The magnetic properties of the layered Ruddlesden–Popper perovskites differ from the ABO_3_ phases as a result of the reduced dimensionality of the magnetic interaction (e.g., nearly two-dimensional for the A_2_BO_4_ case).^57^ Perovskite manganites are model systems of magnetic transition metal oxides. Conventional ABO_3_, A_2_BO_4_, and A_3_B_2_O_7_ manganite phases display rich electronic phase diagrams associated with the stability and instability of spin and orbital orders associated with a given electron/hole concentration (i.e., chemical composition).^58^ As a result, short-range magnetic states and spin glass states appear in the electronic phase diagrams of those materials,^56,58,59^ e.g., for the A_3_B_2_O_7_ manganites.^58,60^ Magnetically frustrated long-range ordered phases may also be stabilized and turn into spin glasses at lower temperatures due to the frustration of the magnetic interaction.^61^ Short-range magnetic order has also been reported for the manganese-containing A_3_B_2_O_7_ phases with (Mn, Co) and (Mn, Fe) on the B sites.^62,63^ While the manganese-containing Ruddlesden–Popper phases have been well studied, there are fewer examples containing multiple other transition metal elements. The reported A_3_B_2_O_7_ phases with multiple transition metals, for example, the Sr_3_Fe_2–*x*_Ni_*x*_O_7−δ_ and Sr_3_Fe_2–*x*_Co_*x*_O_7−δ_ compounds, also display spin glass-like behavior though with evidence of inhomogeneity in the latter case.^64,65^ While spin glass-like behavior is relatively common in perovskites, the effect has not been previously reported in compositionally complex perovskites.

## 4 Conclusions

A new series of compositionally complex perovskites with a general formula of La_0.5_Sr_2.5_(M)_2_O_7−δ_ have been reported. The compounds were synthesized via the solid-state method and analyzed by powder X-ray diffraction. Neutron diffraction experiments on La_0.5_Sr_2.5_(Ti_1/5_Mn_1/5_Fe_1/5_Co_1/5_Ni_1/5_)_2_O_7−δ_ did not reveal any long-range ordering of the cations. The refined oxygen site occupancies revealed oxygen vacancies localized predominantly on the 2*a* position. Magnetometry results indicate that the magnetic properties are qualitatively similar. The temperature dependencies of the dc magnetization and ac susceptibility in these compounds suggest that they undergo spin glass transitions at low temperatures. Although such glassy phases are ubiquitous in the electronic phase diagrams of conventional perovskites and layered perovskites, they have not been previously reported in compositionally complex perovskites.

## Supporting Information Available

Refined parameters from XRD Rietveld refinements, EDS analysis of all compositions, neutron diffraction data of La_0.5_Sr_2.5_(Ti_1/5_Mn_1/5_Fe_1/5_Co_1/5_Ni_1/5_)_2_O_7−δ_ from Polaris bank 4 and from the MEREDIT instrument, neutron diffraction data of La_0.5_Sr_2.5_(Mn_1/4_Fe_1/4_Co_1/4_Ni_1/4_)_2_O_7−δ_, XPS survey spectra of La_0.5_Sr_2.5_(Mn_1/4_Fe_1/4_Co_1/4_Ni_1/4_)_2_O_7−δ_, and magnetometry data for all compositions (PDF)
