# Supplementary material for: Phase Stability and Magnetic Properties of Compositionally Complex n = 2 Ruddlesden–Popper Perovskites
Source: Inorg Chem. 2024 Apr 3;63(15):6616–25. doi: 10.1021/acs.inorgchem.3c04277 (PMC11022176; doi:10.1021/acs.inorgchem.3c04277)
Supplement: Supplementary file 1 — ic3c04277_si_001.pdf [file ic3c04277_si_001.pdf]

Supporting Information for Phase Stability and Magnetic Properties of Compositionally Complex *n* = 2 Ruddlesden-Popper Perovskites

Rebecca Clulow[a*], Prativa Pramanik[b], Amanda Stolpe[a,c], Deep C. Joshi[b], Roland Mathieu[b], Paul F. Henry[a,d] and Martin Sahlberg[a]

[a]*Department of Chemistry – Ångström Laboratory, Uppsala University, Box 538, 751 21, Uppsala, Sweden*
[b]*Department of Materials Science and Engineering, Uppsala University, Box 35, 751 03, Uppsala, Sweden*
[c]*FSCN Research Centre, Surface and Colloid Engineering, Mid Sweden University, 851 70, Sundsvall, Sweden*
[d]*ISIS Pulsed Neutron & Muon Facility, Rutherford Appleton Laboratory, Harwell Campus, OX11 0QX, United Kingdom*

rebecca.clulow@kemi.uu.se

1. X-ray diffraction data

Table S1: Refined atomic coordinates and occupancies of $La_{0.5}Sr_{2.5}(M)_2O_{7-\delta}$ derived from powder X-ray diffraction data collected at room temperature.

	Wyckoff Position	x	y	z	Occupancy	Beq
$La_{0.5}Sr_{2.5}(Ti_{1/5}Mn_{1/5}Fe_{1/5}Co_{1/5}Ni_{1/5})_2O_{7-\delta}$						
La1	2*b*	0	0	0.5	1/6	0.2000
Sr1	2*b*	0	0	0.5	5/6	0.2000
La2	4*e*	0	0	0.3176(1)	1/6	0.2000
Sr2	4*e*	0	0	0.3176(1)	5/6	0.2000
Ti	4e	0	0	0.0987(2)	1/5	0.2000
Mn	4*e*	0	0	0.0987(2)	1/5	0.2000
Fe	4*e*	0	0	0.0987(2)	1/5	0.2000
Co	4*e*	0	0	0.0987(2)	1/5	0.2000
Ni	4*e*	0	0	0.0987(2)	1/5	0.2000
O1	8*g*	0	0.5	0.0872(4)	1	0.2000
O2	4*e*	0	0	0.2002(5)	1	0.2000
O3	2*a*	0	0	0	1	0.2000
$La_{0.5}Sr_{2.5}(Mn_{1/4}Fe_{1/4}Co_{1/4}Ni_{1/4})_2O_{7-\delta}$						
La1	2*b*	0	0	0.5	1/6	0.2000
Sr1	2*b*	0	0	0.5	5/6	0.2000
La2	4*e*	0	0	0.3177(1)	1/6	0.2000
Sr2	4*e*	0	0	0.3177(1)	5/6	0.2000
Mn	4*e*	0	0	0.0995(1)	1/4	0.2000
Fe	4*e*	0	0	0.0995(1)	1/4	0.2000
Co	4*e*	0	0	0.0995(1)	1/4	0.2000
Ni	4*e*	0	0	0.0995(1)	1/4	0.2000
O1	8*g*	0	0.5	0.0911(4)	1	0.2000
O2	4*e*	0	0	0.1995(4)	1	0.2000
O3	2*a*	0	0	0	1	0.2000
$La_{0.5}Sr_{2.5}(Mn_{1/5}Fe_{2/5}Co_{1/5}Ni_{1/5})_2O_{7-\delta}$						
La1	2*b*	0	0	0.5	1/6	0.2000
Sr1	2*b*	0	0	0.5	5/6	0.2000
La2	4*e*	0	0	0.3173(1)	1/6	0.2000
Sr2	4*e*	0	0	0.3173(1)	5/6	0.2000
Mn	4*e*	0	0	0.0992(2)	1/5	0.2000

Fe	4*e*	0	0	0.0992(2)	2/5	0.2000
Co	4*e*	0	0	0.0992(2)	1/5	0.2000
Ni	4*e*	0	0	0.0992(2)	1/5	0.2000
O1	8*g*	0	0.5	0.0901(4)	1	0.3318
O2	4*e*	0	0	0.1981(5)	1	0.3318
O3	2*a*	0	0	0	1	0.3318

$La_{0.5}Sr_{2.5}(Mn_{2/6}Fe_{2/6}Co_{1/6}Ni_{1/6})_2O_{7-\delta}$						
La1	2*b*	0	0	0.5	1/6	0.2000
Sr1	2*b*	0	0	0.5	5/6	0.2000
La2	4*e*	0	0	0.3183(1)	1/6	0.2000
Sr2	4*e*	0	0	0.3183(1)	5/6	0.2000
Mn	4*e*	0	0	0.0987(2)	2/6	0.2000
Fe	4*e*	0	0	0.0987(2)	2/6	0.2000
Co	4*e*	0	0	0.0987(2)	1/6	0.2000
Ni	4*e*	0	0	0.0987(2)	1/6	0.2000
O1	8*g*	0	0.5	0.0900(4)	1	0.2000
O2	4*e*	0	0	0.1988(5)	1	0.2000
O3	2*a*	0	0	0	1	0.2000

2. Scanning electron microscopy data

Table S2: Elemental composition of $La_{0.5}Sr_{2.5}(Ti_{1/5}Mn_{1/5}Fe_{1/5}Co_{1/5}Ni_{1/5})_2O_{7-\delta}$ from EDS measurements. Values given in at%.

Spectrum	La	Sr	Ti	Mn	Fe	Co	Ni
1	10.81	49.31	8.76	8.23	8.41	7.76	6.73
2	9.85	52.85	7.97	7.91	7.50	7.24	6.68
3	10.45	50.87	8.08	8.75	7.57	7.30	6.98
4	10.42	49.00	8.42	8.95	8.36	7.92	6.92
5	9.59	51.58	7.96	8.21	8.02	7.65	7.00
6	10.52	50.53	8.59	8.20	7.71	7.26	7.19
7	10.20	52.95	7.76	8.22	6.92	7.41	6.54
8	10.02	52.99	7.94	7.53	7.68	7.22	6.62
9	10.32	51.82	7.72	7.92	7.89	7.46	6.86
10	9.97	51.57	7.83	8.14	8.02	7.75	6.71

Statistic	La	Sr	Ti	Mn	Fe	Co	Ni
Max	10.81	52.99	8.76	8.95	8.41	7.92	7.19
Min	9.59	49.00	7.72	7.53	6.92	7.22	6.54
Average	10.22	51.35	8.10	8.21	7.81	7.50	6.82
Standard Deviation	0.36	1.43	0.36	0.41	0.44	0.25	0.20

Table S3: Elemental composition of $La_{0.5}Sr_{2.5}(Mn_{1/4}Fe_{1/4}Co_{1/4}Ni_{1/4})_2O_{7-\delta}$ from EDS measurements. Values given in at%.

Spectrum	La	Sr	Mn	Fe	Co	Ni
1	11.02	51.36	10.75	9.97	9.37	7.53

2	11.35	46.57	11.24	11.14	11.28	8.42
3	11.40	48.05	11.16	10.58	10.75	8.06
4	13.34	36.88	13.46	13.45	12.79	10.08
5	11.59	46.59	11.47	11.46	10.78	8.11
6	11.48	47.85	11.25	10.98	10.67	7.78
7	12.13	47.75	10.91	10.67	10.22	8.33
8	13.19	38.97	12.68	13.09	12.67	9.39
9	12.55	43.57	12.09	11.42	11.43	8.94
10	11.09	50.18	10.58	10.27	9.83	8.04

Statistic	La	Sr	Mn	Fe	Co	Ni
Max	13.34	51.36	13.46	13.45	12.79	10.08
Min	11.02	36.88	10.58	9.97	9.37	7.53
Average	11.91	45.78	11.56	11.30	10.98	8.47
Standard Deviation	0.85	4.66	0.92	1.14	1.11	0.78

Table S4: Elemental composition of $La_{0.5}Sr_{2.5}(Mn_{1/5}Fe_{2/5}Co_{1/5}Ni_{1/5})_2O_{7-\delta}$ from EDS measurements. Values given in at%.

Spectrum	La	Sr	Mn	Fe	Co	Ni
1	11.09	45.38	8.95	17.84	8.56	8.17
2	10.86	51.22	7.99	15.55	7.33	7.05
3	10.74	51.26	8.43	15.58	7.11	6.89
4	10.28	51.84	8.34	15.68	7.12	6.74
5	10.38	52.70	7.91	15.22	7.17	6.62
6	10.55	51.01	7.59	16.28	7.62	6.95
7	10.84	50.29	8.12	16.16	7.74	6.85
8	11.00	48.85	8.42	16.49	8.11	7.14
9	10.92	48.16	8.02	16.89	8.36	7.65
10	11.48	45.18	9.15	17.71	8.69	7.79
11	10.48	51.75	8.02	15.46	7.51	6.78

Statistic	La	Sr	Mn	Fe	Co	Ni
Max	11.48	52.7	9.15	17.84	8.69	8.17
Min	10.28	45.18	7.59	15.22	7.11	6.62
Average	10.78	49.78	8.27	16.26	7.76	7.15
Standard Deviation	0.35	2.58	0.46	0.90	0.59	0.50

Table S5: Elemental compositions of $La_{0.5}Sr_{2.5}(Mn_{2/6}Fe_{2/6}Co_{1/6}Ni_{1/6})_2O_{7-\delta}$ from EDS measurements. Values given in at%.

Spectrum	La	Sr	Mn	Fe	Co	Ni
1	10.78	51.38	12.82	12.51	6.55	5.96
2	11.67	50.46	12.39	13.08	6.41	5.98
3	11.12	49.17	14.04	13.17	6.40	6.09
4	10.58	50.61	13.70	12.59	6.25	6.27
5	11.10	46.85	15.04	13.98	6.55	6.48

6	10.23	52.21	13.46	12.28	6.00	5.81
7	10.74	49.65	14.03	12.76	6.56	6.26
8	10.83	50.39	13.85	12.74	6.35	5.85
9	10.85	49.53	14.33	13.31	6.07	5.91
10	10.98	48.46	14.53	13.33	6.24	6.47
11	8.39	61.67	10.37	10.04	5.08	4.44

Statistic	La	Sr	Mn	Fe	Co	Ni
Max	11.67	61.67	15.04	13.98	6.56	6.48
Min	8.39	46.85	10.37	10.04	5.08	4.44
Average	10.66	50.94	13.51	12.71	6.22	5.96
Standard Deviation	0.83	3.84	1.28	1.00	0.42	0.56

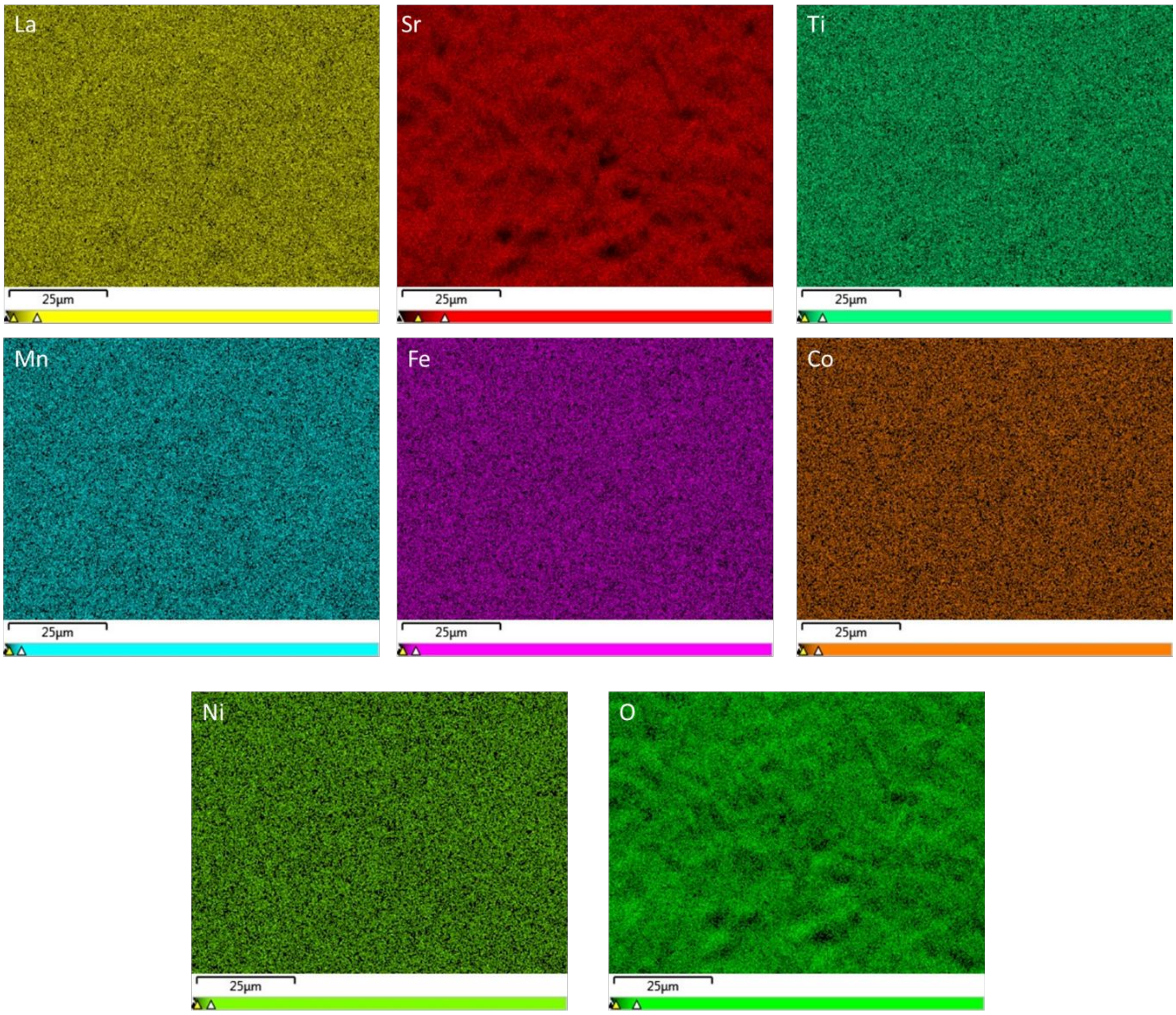

Figure S1: EDS Mapping images taken on the surface of a sintered pellet of $La_{0.5}Sr_{2.5}(Ti_{1/5}Mn_{1/5}Fe_{1/5}Co_{1/5}Ni_{1/5})_2O_{7-\delta}$.

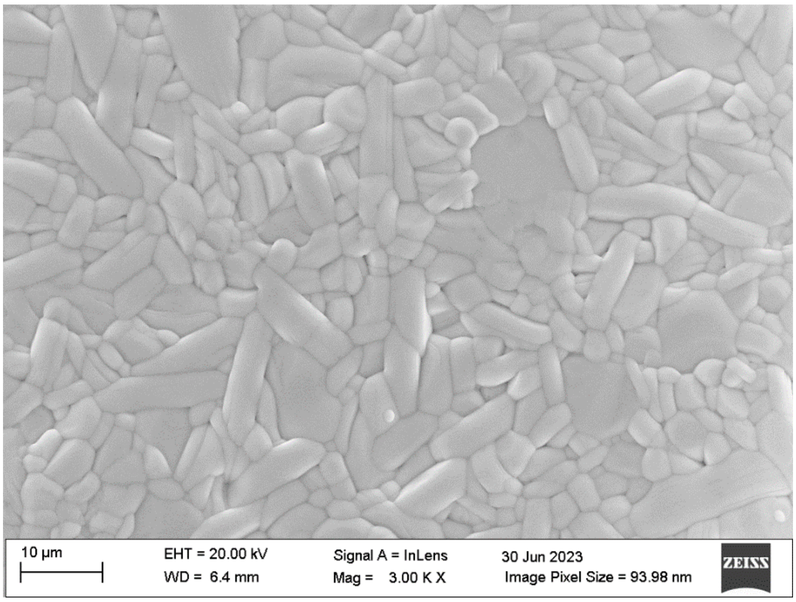

Figure S2: SEM image taken on the surface of a sintered pellet of $La_{0.5}Sr_{2.5}(Ti_{1/5}Mn_{1/5}Fe_{1/5}Co_{1/5}Ni_{1/5})_2O_{7-\delta}$ showing the area used for EDS mapping.

3. Neutron diffraction data for $La_{0.5}Sr_{2.5}(Ti_{1/5}Mn_{1/5}Fe_{1/5}Co_{1/5}Ni_{1/5})_2O_{7-\delta}$

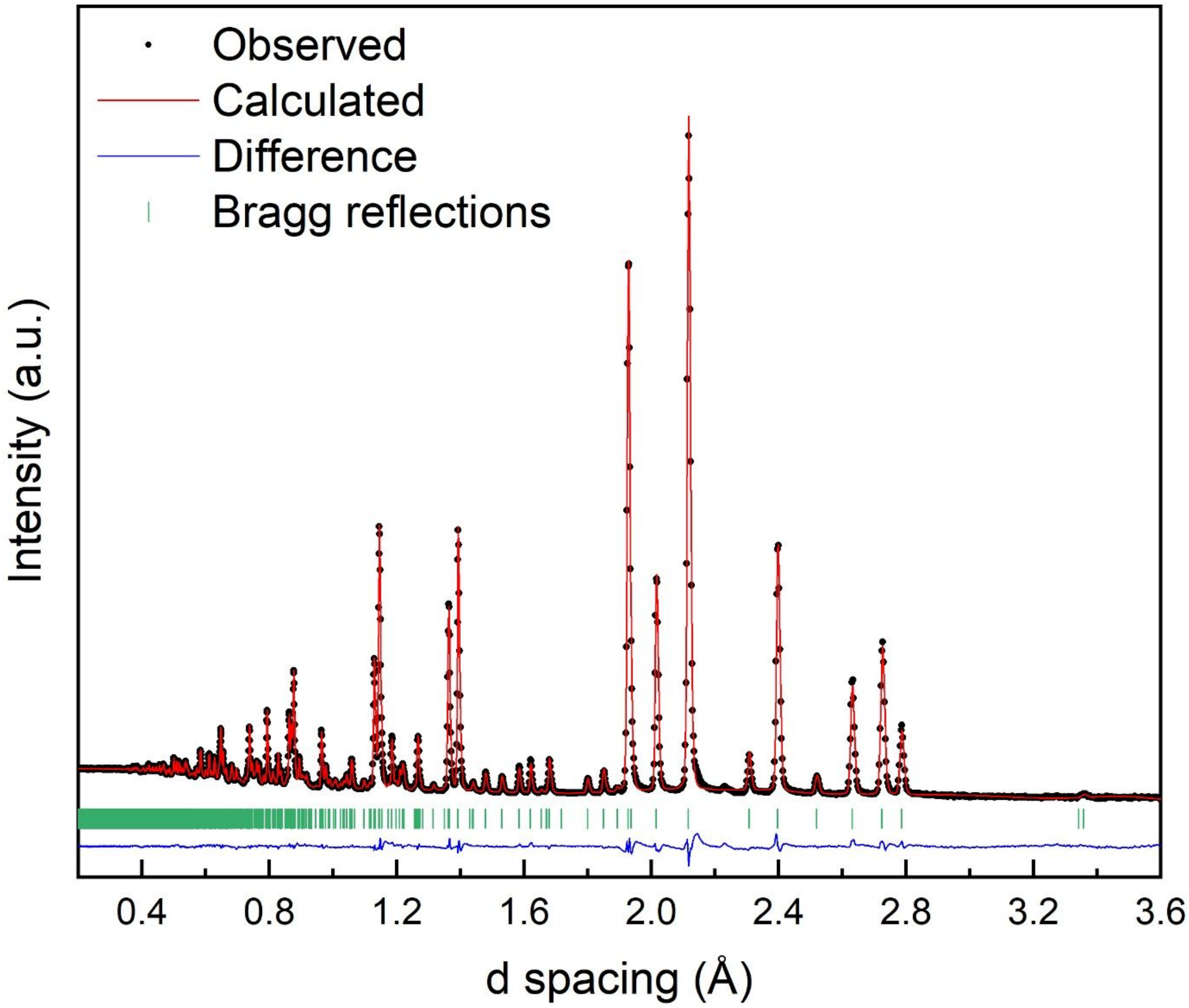

Figure S3: Powder neutron diffraction patterns and Rietveld refinement of data collected from the 4th detector bank on the Polaris powder diffractometer at the ISIS neutron and muon source $La_{0.5}Sr_{2.5}(Ti_{1/5}Mn_{1/5}Fe_{1/5}Co_{1/5}Ni_{1/5})_2O_{7-\delta}$ R_{wp} 2.32 χ^2 21.91.

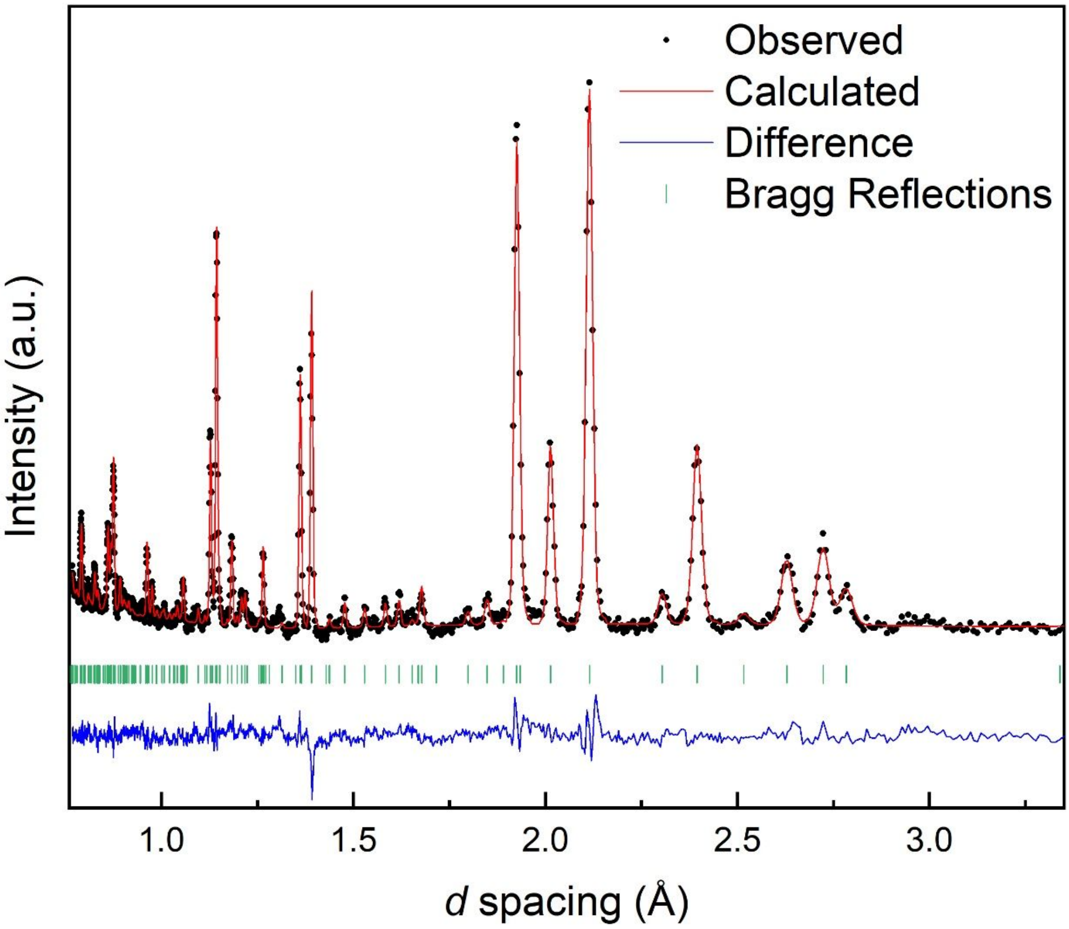

Figure S4: Powder neutron diffraction patterns and Rietveld refinements of $La_{0.5}Sr_{2.5}(Ti_{1/5}Mn_{1/5}Fe_{1/5}Co_{1/5}Ni_{1/5})_2O_{7-\delta}$ R_{wp} 5.04 χ^2 3.44 from data collected using the MEREDIT diffractometer. Refined unit cell parameters *a* = 3.8507(2) and *c* = 20.1348(14) Å.

Table S6: Refined atomic positions and occupancies of $La_{0.5}Sr_{2.5}(TiMnFeCoNi)_2O_{7-\delta}$ derived from powder neutron diffraction data collected at room temperature using the MEREDIT diffractometer.

Atom	Wyckoff Position	*x*	*y*	*z*	Occupancy	Uiso × 100
La	2*b*	0	0	0.5	1/6	0.931(0.172)
Sr	2*b*	0	0	0.5	5/6	0.931(0.172)
La	4*e*	0	0	0.3182(2)	1/6	0.558(0.122)
Sr	4*e*	0	0	0.3182(2)	5/6	0.558(0.122)
M	4*e*	0	0	0.0982(7)	1	2.932(0.286)
O1	8*g*	0	0.5	0.0937(2)	0.958(22)	1.094(0.121)
O2	4*e*	0	0	0.1957(3)	1	0.678(0.192)
O3	2*a*	0	0	0	0.780(28)	0.129(0.307)

4. Neutron diffraction data for $La_{0.5}Sr_{2.5}(Mn_{1/4}Fe_{1/4}Co_{1/4}Ni_{1/4})_2O_{7-\delta}$

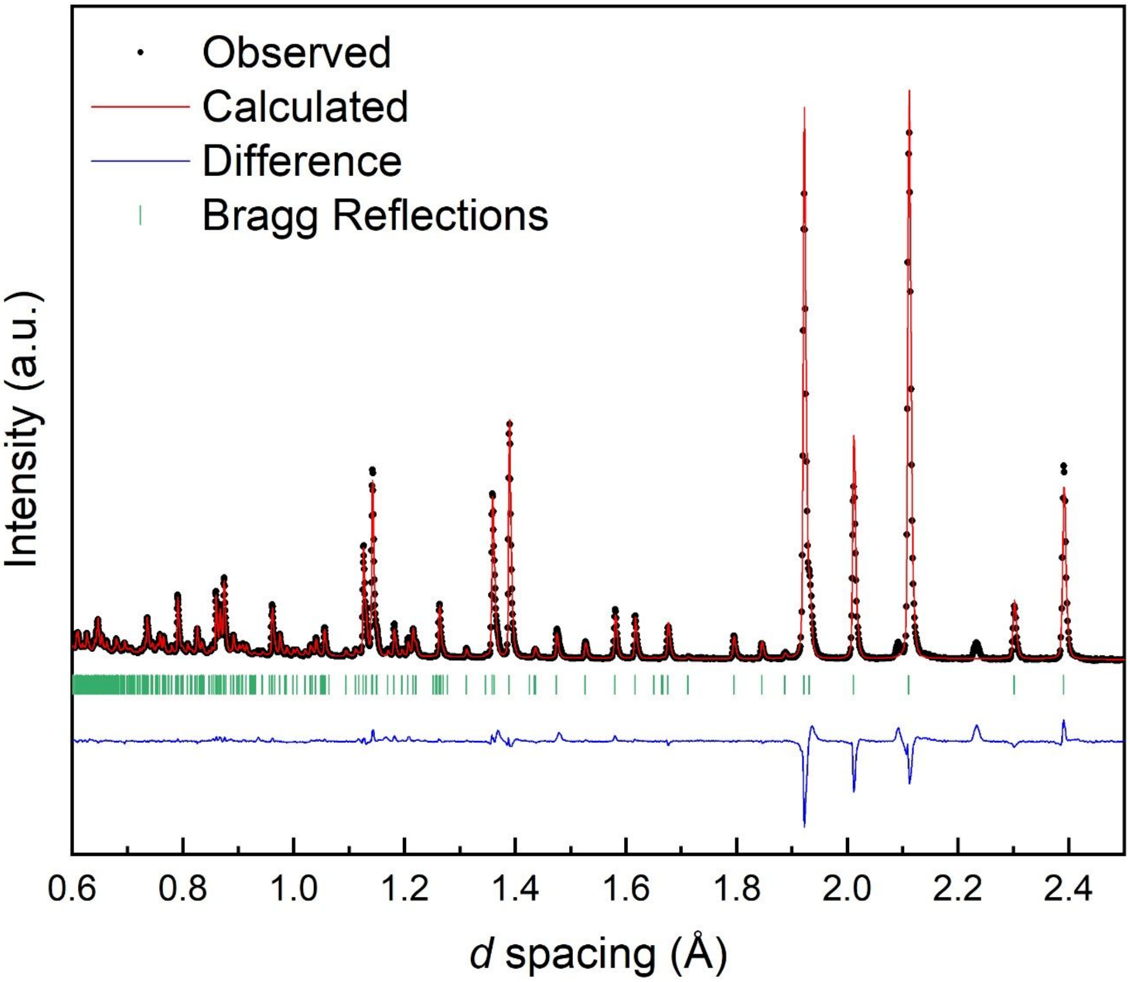

Figure S5: Powder neutron diffraction patterns and Rietveld refinement of $La_{0.5}Sr_{2.5}(Mn_{1/4}Fe_{1/4}Co_{1/4}Ni_{1/4})_2O_{7-\delta}$ R_{wp} 4.09 χ^2 25.64 from data collected using the Polaris diffractometer. Refined unit cell parameters *a* = 3.8423(3) and *c* = 20.1068(15) Å.

Table S7: Refined atomic positions and occupancies of $La_{0.5}Sr_{2.5}(Mn_{1/4}Fe_{1/4}Co_{1/4}Ni_{1/4})_2O_{7-\delta}$ derived from powder neutron diffraction data collected at room temperature using the Polaris diffractometer.

Atom	Wyckoff Position	*x*	*y*	*z*	Occupancy	Uiso × 100
La	2*b*	0	0	0.5	1/6	0.725(0.037)
Sr	2*b*	0	0	0.5	5/6	0.725(0.037)
La	4*e*	0	0	0.3181(1)	1/6	0.502(0.024)
Sr	4*e*	0	0	0.3181(1)	5/6	0.502(0.024)
M	4*e*	0	0	0.0993(1)	1	0.329(0.031)
O1	8*g*	0	0.5	0.0925(1)	0.94(1)	0.540(0.034)
O2	4e	0	0	0.1948(1)	0.98(1)	0.678(0.038)
O3	2*a*	0	0	0	0.72(2)	0.850(0.103)

5. X-ray photoelectron spectroscopy

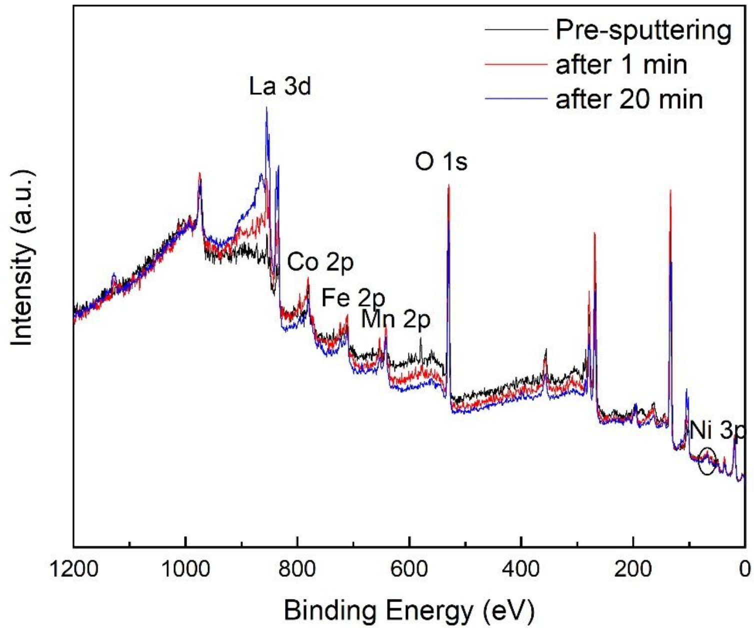

Figure S6: Survey spectra from X-ray photoelectron spectroscopy measurements pre and post sputtering with regions used in further analysis marked.

6. Magnetometry

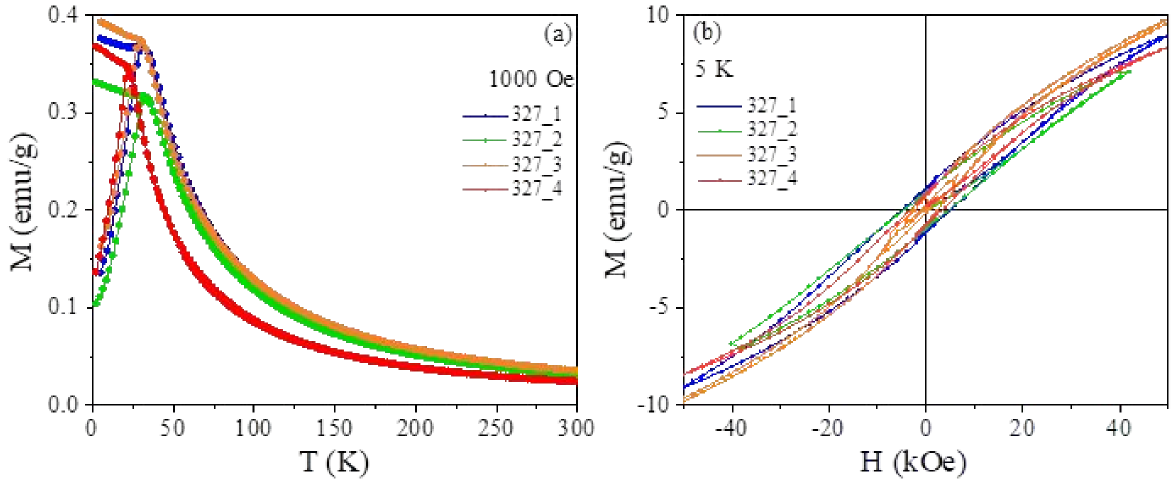

Figure S7: (a) ZFC/FC magnetization M vs. T recorded in H = 1000 Oe and (b) M vs. H at 5K for all samples. 327_1: $La_{0.5}Sr_{2.5}(Mn_{1/4}Fe_{1/4}Co_{1/4}Ni_{1/4})_2O_{7-\delta}$; 327_2: $La_{0.5}Sr_{2.5}(Mn_{1/5}Fe_{2/5}Co_{1/5}Ni_{1/5})_2O_{7-\delta}$; 327_3: $La_{0.5}Sr_{2.5}(Mn_{2/6}Fe_{2/6}Co_{1/6}Ni_{1/6})_2O_{7-\delta}$; 327_4: $La_{0.5}Sr_{2.5}(Ti_{1/5}Mn_{1/5}Fe_{1/5}Co_{1/5}Ni_{1/5})_2O_{7-\delta}$.
